# Associations between Health-Related Quality of Life, Illness Perception, Stigmatization and Optimism among Hematology Patients: a Path Analysis

**DOI:** 10.1192/j.eurpsy.2024.445

**Published:** 2024-08-27

**Authors:** H. Kiss, V. Müller, K. T. Dani, B. F. Pikó

**Affiliations:** ^1^Department of Behavioral Sciences; ^2^Doctoral School of Education; ^3^Department of Hematology, Department of Internal Medicine, University of Szeged, Szeged, Hungary

## Abstract

**Introduction:**

Hematological diseases represent a diverse disease group ranging from benign to life-threatening conditions, with hematological malignancies being a major cause of mortality in the population worldwide. Although most hematological diseases require ongoing medical care making these conditions even more difficult for patients to endure. Since these diseases can pose many challenges by causing symptoms and limitations in various aspects of daily life, health-related quality of life (HRQoL) is a crucial aspect of their healthcare. Different dimensions of health-related quality of life are influenced by several psychological factors, including illness perception, stigmatization, and optimism: a more positive illness perception, along with optimism and reduced stigmatization, can contribute to a better HRQoL among hematology patients.

**Objectives:**

Since hematological diseases often cause serious life changes, the current study aimed to explore the direct and indirect effects of illness perception on health-related quality of life among hematology patients in Hungary, including stigmatization and optimism as possible contributors.

**Methods:**

In this cross-sectional study, 96 hematology patients (mean age = 56.45 years; SD = 15.55 years; 43.8% female) completed a self-administered survey including the following instruments: EORTC Quality of Life Scale, Brief Illness Perception Questionnaire, Stigma Scale for Chronic Illness, Revised Life Orientation Test.

**Results:**

By creating two pathway models, illness perception had significant indirect effects on physical functioning (β = -.205, p < .05) through role and cognitive functioning while emotional functioning had significant indirect effects on social functioning (β = .369, p < .01) through illness perception and stigmatization, both effects moderated by optimism. After controlling for other factors, both illness perception and emotional functioning directly influenced physical and social functioning, respectively.

**Conclusions:**

Our study supports previous research on the direct and indirect effects of illness perception on HRQoL. Based on our data, more optimistic illness perceptions and greater emotional functioning improve hematology patients’ health-related quality of life by facilitating an unbiased understanding of the disease. Optimism serves as a potential moderating mechanism by positively altering indirect effects. Healthcare professionals need to optimize patients’ illness perception to improve physical and social functioning.

**Disclosure of Interest:**

H. Kiss Grant / Research support from: This work was supported by the New National Excellence Program of the Ministry for Culture and Innovation from the source of the National Research, Development and Innovation Fund, #ÚNKP-22-4-SZTE-301., V. Müller: None Declared, K. Dani: None Declared, B. Pikó: None Declared

